# Pigmentation and not only sex and age of individuals affects despotism in the Andean condor

**DOI:** 10.1371/journal.pone.0205197

**Published:** 2018-10-24

**Authors:** Nancy V. Marinero, Verónica B. Cailly-Arnulphi, Sergio A. Lambertucci, Carlos E. Borghi

**Affiliations:** 1 Universidad Nacional de San Juan - CIGEOBIO CONICET, San Juan, Argentina; 2 Grupo de Investigaciones en Biología de la Conservación, Laboratorio Ecotono, Universidad Nacional del Comahue - INIBIOMA CONICET, Bariloche, Río Negro, Argentina; 3 Departamento de Biología y Museo de Ciencias Naturales, Facultad de Ciencias Exactas, Físicas y Naturales, Universidad Nacional de San Juan, San Juan, Argentina; University of Lleida, SPAIN

## Abstract

Attributes such as sex, age and pigmentation of individuals could correspond to the competitive skills they use to access resources and, consequently, determine their social status when a hierarchy of dominance is established. We analysed patterns of social dominance in relation to sex, age and, for the first time, according to face pigmentation in a large scavenger bird species, the Andean condor (*Vultur gryphus*). This species displays extreme sexual dimorphism, with males being up to 50% heavier than females. Associated to this, strong hierarchical relationships characterize foraging, roosting and breeding. We recorded agonistic interactions within condor groups while foraging through video recordings in experimental stations. We corroborated a strong despotism by the adult males to the rest of the categories. More interestingly we found this despotism was also expressed by most pigmented birds; juvenile females being completely subordinated and, at the same time, not expressing pigmentation. Importantly, when condors of equal sex and age category fought, the more pigmented individuals were successful. Our results highlight that pigmentation, besides sex and age, is an attribute that also corresponds with social status in the Andean condor, making its hierarchical system more complex.

## Introduction

Feeding in groups is a foraging strategy that brings numerous benefits to group members, such as increasing the likelihood of locating food and enhancing vigilance for predator detection [1]. However, this social behavior also increases the likelihood of individuals competing for food resources [2]. Such competition involves great cost to those individuals engaging in fights because of the expenditure of time and energy involved and possible injuries [3].

In agonistic interactions, successful resource acquisition by individuals depends on the characteristics or attributes of each forager [4]. According to the “phenotypic limitation” model, there exists an asymmetry in the competitive abilities of individuals that correlates with attributes such as age and body size [5]. This implies that, in the presence of interindividual differences, older and/or individuals with large body size, tend to be more dominant and benefit mainly through higher intake rates [2], [6]. Also, the relative pay-offs according to body size are generally correlated with sex in species with dimorphism. In birds, usually males are the individuals most benefited by having a greater body size [7], [8], [9].

Another signal of social status can be the pigmentation of individuals. Particularly, in many birds, pigmentation of plumage and skin is an attribute indicative of their social status [8]. Some colors like red, orange and yellow, typically of long duration, are generally achieved through chemical compounds called carotenoids that cannot be synthesized directly by the individuals, so they are only actively incorporated from the environment through a diet rich in plants [10]. This micronutrient has important health functions mainly related to immune responses, antioxidants and reproduction, therefore generating a trade-off in carotenoid allocation that ensures the “honesty” of bright pigmentation as a condition-dependent trait: only superior quality individuals can afford pigmentation without compromising their health [11], [12]. Moreover, there is a taxonomically diverse group of birds that exhibit rapid changes in the intensity of the coloration of highly vascularized featherless skin patches, called “facial flushing”, produced by the variation in blood flow through the tissue [13]. Within a competition context, the difference in the intensity of carotenoid-dependent pigmentation and facial flushing increases between dominant and subordinates reflecting limitations in access to resources for subordinates and/or variation in the life requirements of individuals depending on their social status [13], [14].

Large scavenger birds are constantly competing for food because carrion is an ephemeral resource heterogeneously distributed in space [15]. The resolution of these conflicts can be influenced by the presence and intensity of the pigmentation exhibited by the opponents [16], [17]. In this work, we studied the largest soaring bird in the world, the Andean condor *Vultur gryphus* [18] which, despite being the heaviest raptor species, showed much higher carotenoid concentrations in wildlife situation, in comparison with other predatory and scavenger raptors [19]. This species is an excellent model to test whether pigmentation plays a key role in hierarchical relationships, since its appearance suggests carotenoid deposition in the tongue, iris and bare skin in the neck and head, including the comb and wattles in males, in which the color intensifies by means of a flushing display [13], [20]. Furthermore, condors display extreme sexual dimorphism, with males being up to 50% heavier than females [9], and are a highly despotic species, with hierarchies determined by sex and age [21], [22].

In this framework, we analyzed dominance hierarchy in relation to sex, age and for the first time, according to pigmentation in the Andean condor. Our hypothesis is that dominance hierarchy is directly affected by sex, age and pigmentation due to the relationship of those variables with the size and health of the individuals. We expect that the large-sized individuals (male adults) as well as those more pigmented, are dominant over small-sized, poorly pigmented individuals when competing for food at carcasses.

## Materials and methods

This study was approved by the Comité de Bioética from the department of Biology of the Universidad Nacional de San Juan, Acta N°17, Exp. 02-3243-C.

### Study area

**Fig 1:**
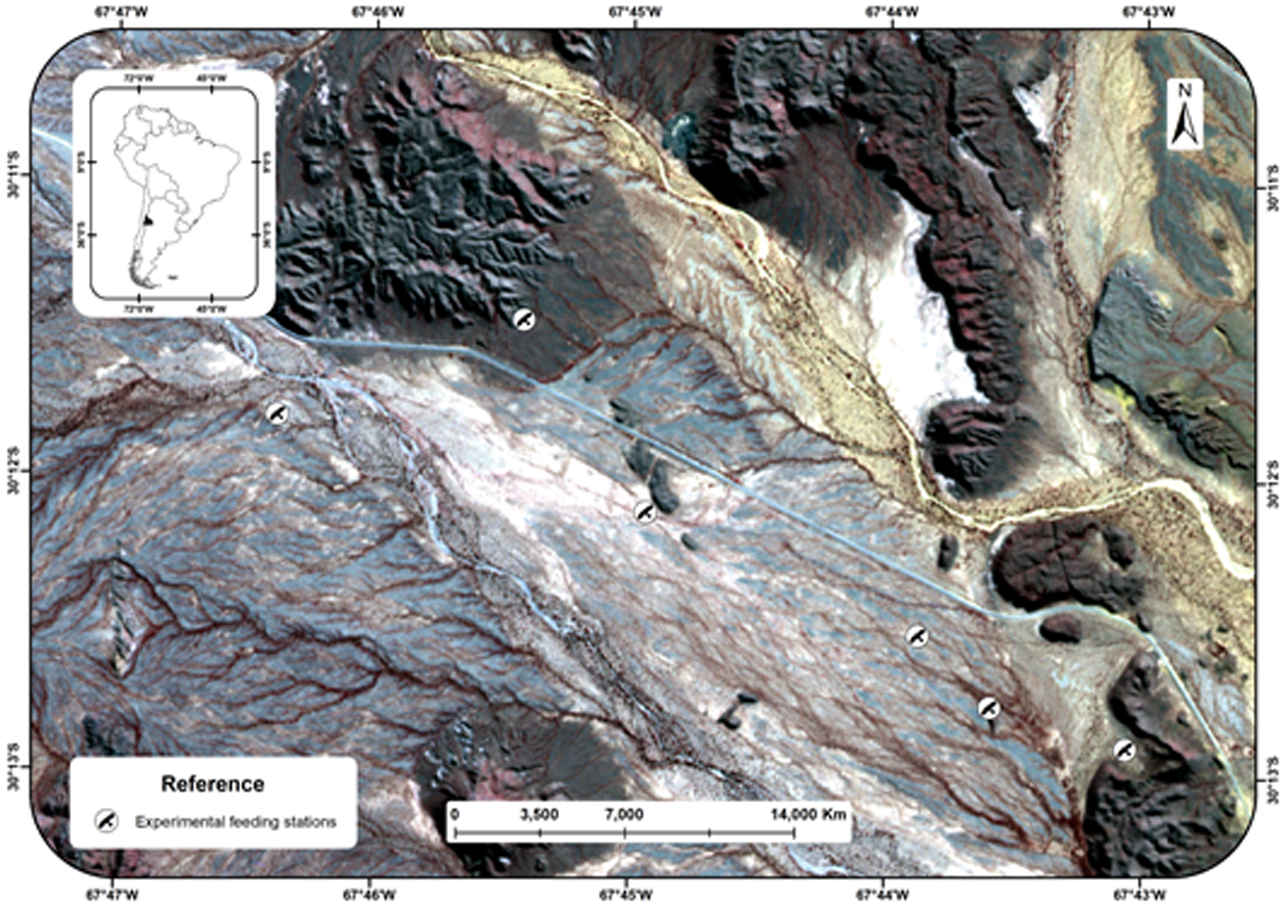
Location of study area in the Valle Fértil department, San Juan province, Argentina (top left) and location of experimental feeding stations.

The study was conducted in the Valle Fértil department, province of San Juan, Argentina (67°44’52” W and 30°11’58” S) (Fig 1). This area corresponds to the ecotone between the Monte of Hills and Closed Basins and Arid Chaco ecoregions [23], [24], the driest area of Argentina, with a mean annual rainfall of around 100 mm [25], whereas mean annual temperature is 22°C [26]. The vegetation is xerophilous and characterized by plant communities that form open shrublands dominated by the shrubs *Larrea cuneifolia* and *Zuccagnia punctacta*, besides there are gallery woodlands of *Prosopis flexuosa* and *Prosopis chilensis* [26]. At the study site, the Andean condor population has a relative abundance, approximately 0.03 ind/km^2^, and up to 45 individuals can be found on a carcass at the same time [27].

### Study species

The Andean condor is the largest soaring bird in the world [18]. In this sexually dimorphic species, individuals aggregate for foraging [28], [29] and roosting [30]. However, from previous studies in other condor distribution areas, it is known that this is a strongly despotic species [23], [31], [32], with adult males being on top of the rank and juvenile females in the lowest rank [22]. In the study area, it is common to observe natural populations of Andean condors that gather in groups to feed on carcasses of domestic (cows and goats) and wild animals (donkeys and guanacos) which represent an ephemeral, unpredictable resource, heterogeneously distributed over space [27]. Local people consider the species to be harmful to livestock, which leads to its being hunted or poisoned [33], [34]. Currently it is considered Nearly Threatened worldwide, and threatened in Argentina [35].

### Sampling design

**Fig 2:**
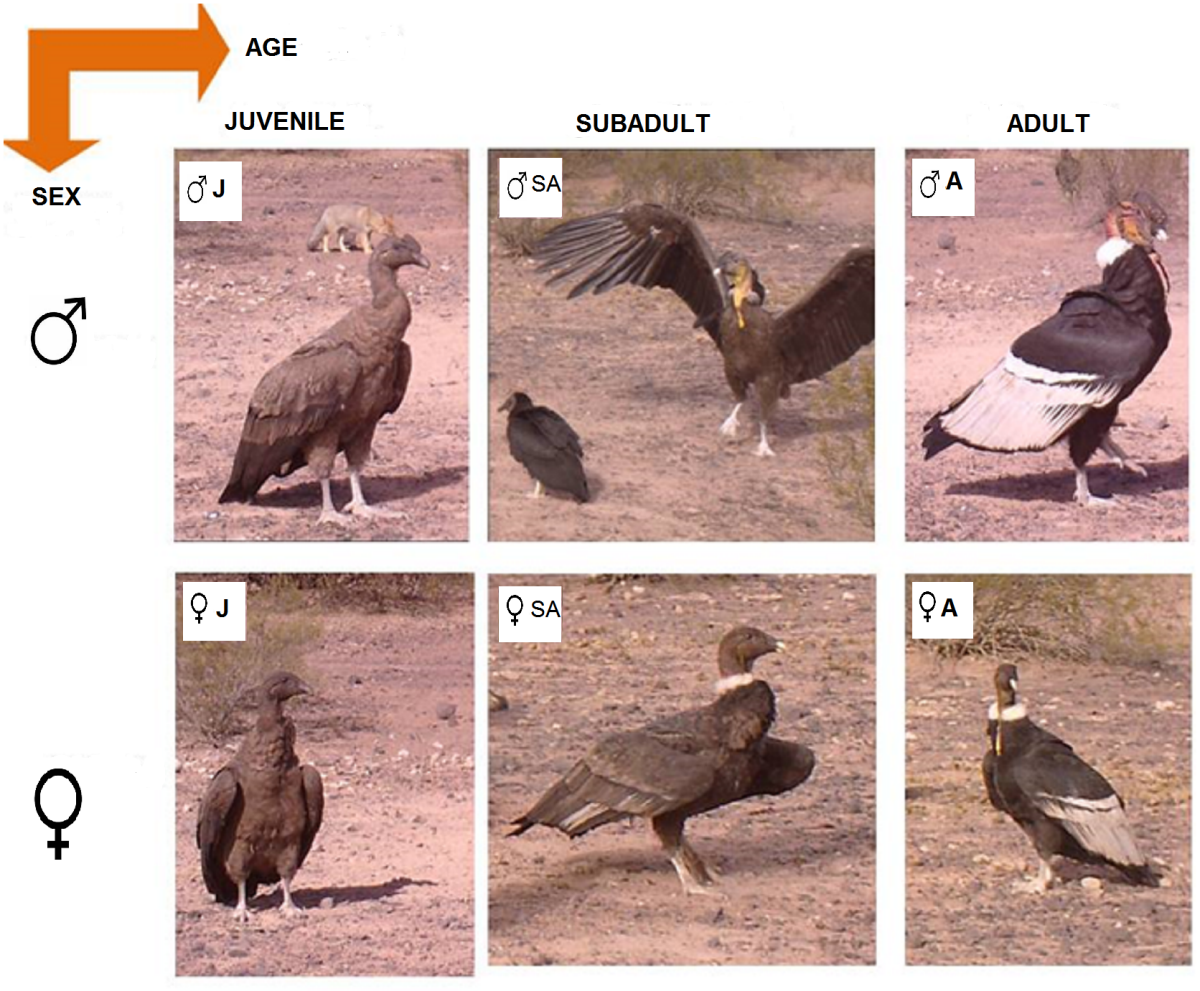
Andean condor categories according to sex and age classes. ♂J: Juvenile male, ♂SA: Sub-adult male, ♂A: Adult male, ♀J: Juvenile female, ♀SA: Sub-adult female, ♀A: Adult female.

**Fig 3:**
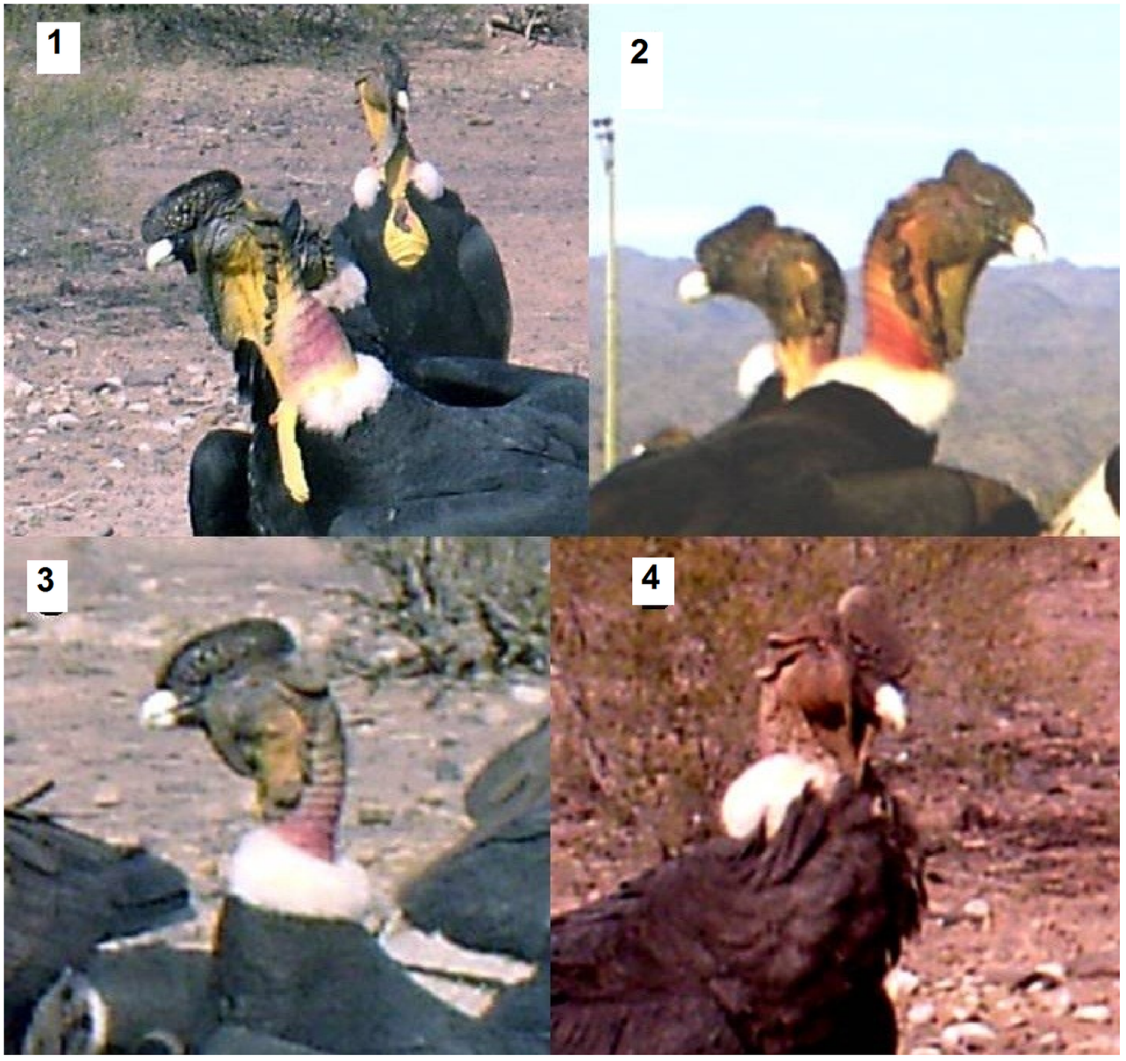
Pigmentation categories in the Andean condor according to presence and intensity of yellow pigmentation. 1) High: head, neck and chest are pigmented and color intensity is high; 2) Medium: head or great part of it, and only part of the neck is pigmented; 3) Low: pigmented patches only on the head and color intensity is low; and 4) Unpigmented: the individual has no yellow pigmentation.

From 2012 to 2015, photographic trapping campaigns were conducted on six sites. At each site, donkey (*Equus asinus*) carcasses (weighing between 150 and 160 kg) were deposited experimentally (six carcasses in total) (Fig 1). In order to record behavior and social structure of condor groups while foraging, videotaping was made at each experimental station. Each videotape lasted 1 minute (maximum shooting time of camera traps) with one-minute videotape intervals without filming. From these records, we systematically selected a video every 10 minutes of filming, to obtain variability in the individuals that visited the carrion and greater independence between records. Subsequently, the following data was recorded from each aggressive interaction: sex and age of individuals, level of pigmentation of the aggressor (initiates the attack), the aggressed (receives the attack), and the affected individual (displaced from carrion) which can be the aggressor or effectively the aggressed. The sex of individuals was established from the presence (male) or absence (female) of comb. Age of each male and female was determined from plumage coloration: juvenile (uniformly brown plumage), sub-adult (brown or gray plumage with white or grayish collar) and adult (black and white plumage with white collar). Then, taking into account that the result of fights can be affected by the abilities that correlate with both the sex and age of condors [21], [22], categories with both attributes were established (Fig 2). Pigmentation level was determined based on presence and intensity of yellow pigmentation: High: head, neck and chest are pigmented, and color intensity is high; Medium: head, or great part of it, and only part of the neck is pigmented; Low: pigmented patches only on the head, and color intensity is low; and Unpigmented: the individual has no yellow pigmentation (Fig 3). Likewise, although the pigmentation could be modified [13], we assumed that the intensity of the pigmentation that condors exhibit at the time of the interaction is the most intense possible, in order to have the greatest possibility of winning the encounter.

### Data analysis

To establish the dominance hierarchy order in feeding groups, frequency matrices were constructed considering, on the one hand, the sex and age categories of condors (Fig 2) and, on the other hand, the categories describing the pigmentation level of individuals (Fig 3). An individual was considered the winner of an encounter when: 1) it starts an attack and displaces or directly expels the other individual from carrion, or 2) it receives the attack but displaces the individual who started the attack. Afterwards, frequency matrices were converted into probability matrices, and then the results were plotted on a graph. We used the R 3.0.2 software (http://www.r-project.org/) to construct the graph of the probability matrix. Moreover, to assess the statistical significance of the frequency of interactions won, we performed a binomial test using the GraphPad software (www.graphpad.com). Then, with the results of both statistical analyses, we determined the dominance relationships. In addition, the probability that an individual wins the fight when exhibiting different levels of pigmentation (more pigmentation or less pigmentation) than the opponent was determined through contingency tables. For this, we used the data on aggressive interactions where individuals had the same sex and age. The level of statistical significance used for all analyses was p ≤ 0.05.

## Results

A total of 468 interactions were recorded in Andean condor feeding groups, of which 282 were aggressions. The greatest number of interactions was initiated by adult males (41.03%), followed by adult females (32.05%), sub-adult males (10.26%), sub-adult females (7.05%) and, finally, by juvenile males and females (5.34% and 4.27%, respectively).

**Fig 4:**
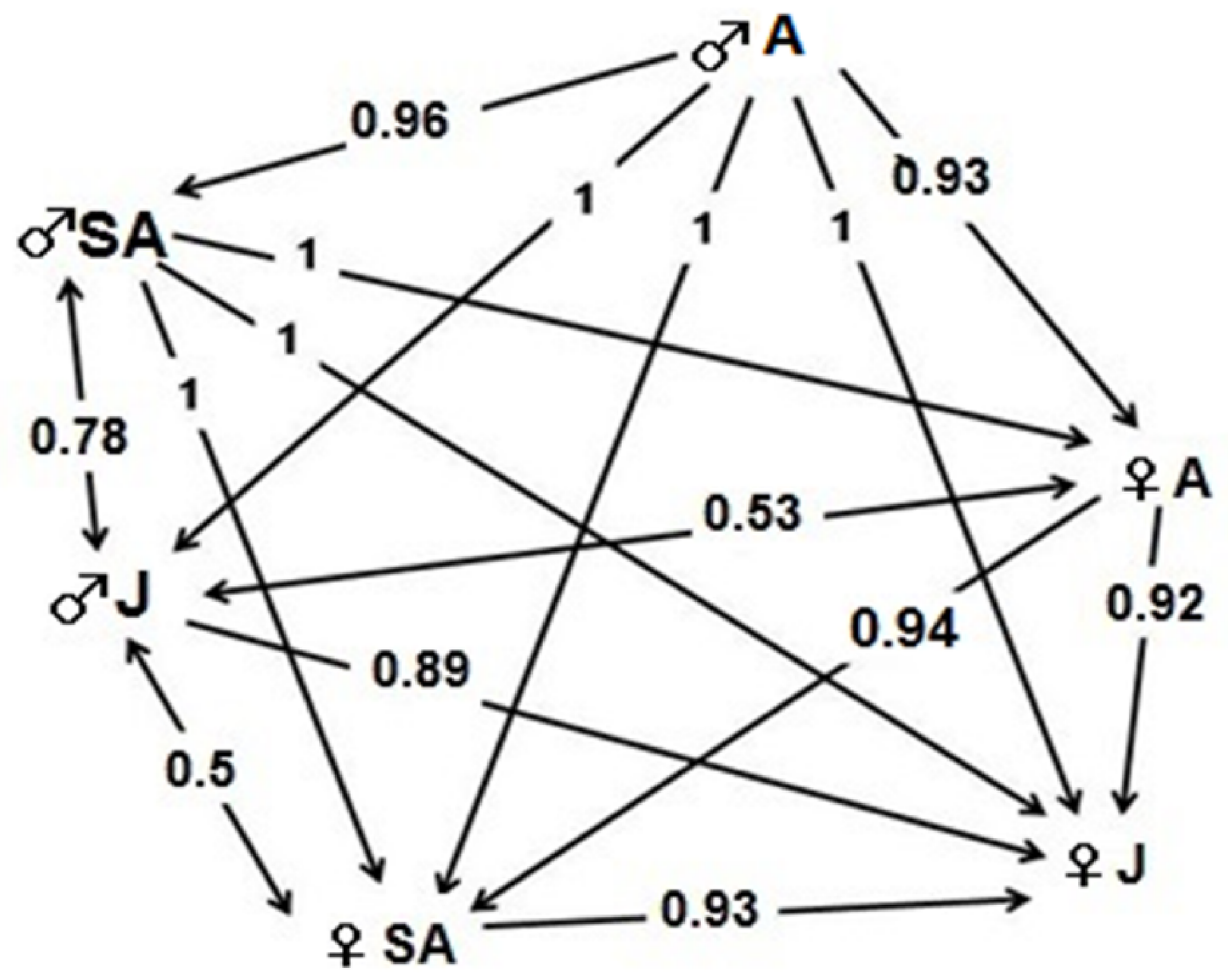
Dominance hierarchies in Andean condor feeding groups, according to sex and age of individuals (N = 282 aggression interactions). Unidirectional arrows indicate the direction of dominance from the dominant individual toward the subordinate one. Double sense arrows indicate interactions between individuals having similar dominance. Values on the arrows indicate likelihood of success and between parenthesis the number of interactions between categories (N).

Attacks by adult and sub-adult males were directed toward all other categories; similarly, juvenile males also attacked nearly all categories, except adult males. Particularly, adult males stand out because, overall, they had a greater likelihood of success in their fights irrespective of the sex and age of the other individual, thus being the most dominant individuals within the hierarchy structure (Table 1 and Fig 4). But dominance becomes diffuse in the intermediate categories in both males and females. In particular, sub-adult males were successful in all fights against females, but not completely successful when the opponents were juvenile males. Besides, juvenile males won against juvenile females, but there were no statistically significant differences in their attacks against sub-adult females (Table 1 and Fig 4). In turn, adult females exhibited interactions with virtually all categories, and were more successful when attacking other sub-adult and juvenile females, but not against juvenile males. Finally, sub-adult females were, overall, more successful in fights against juvenile females, which occupy the other extreme of the hierarchy for being totally subordinated by the other categories (Table 1 and Fig 4).

**Table 1:** Interactions in Andean condor feeding groups according to sex and age of individuals (N = 282 aggression interactions). The level of statistical significance for the binomial test was set at p ≤ 0.05.

Interactions		p-value
♂ A	♀A	<0.0001
♂ A	♂SA	<0.0001
**♂** A	♂J	<0.0001
**♂** A	♀SA	0.0300
**♂** A	♀J	0.0020
**♂** SA	♀A	0.0001
♂ SA	♀SA	0.0078
♂ SA	♀J	0.0078
♂ SA	♂J	0.0890
♂ J	♀J	0.0500
♂ J	♀SA	0.7500
♀ A	♀SA	0.0001
♀ A	♀J	0.0001
♀ A	♂J	0.5000
♀ SA	♀J	0.0009

**Fig 5:**
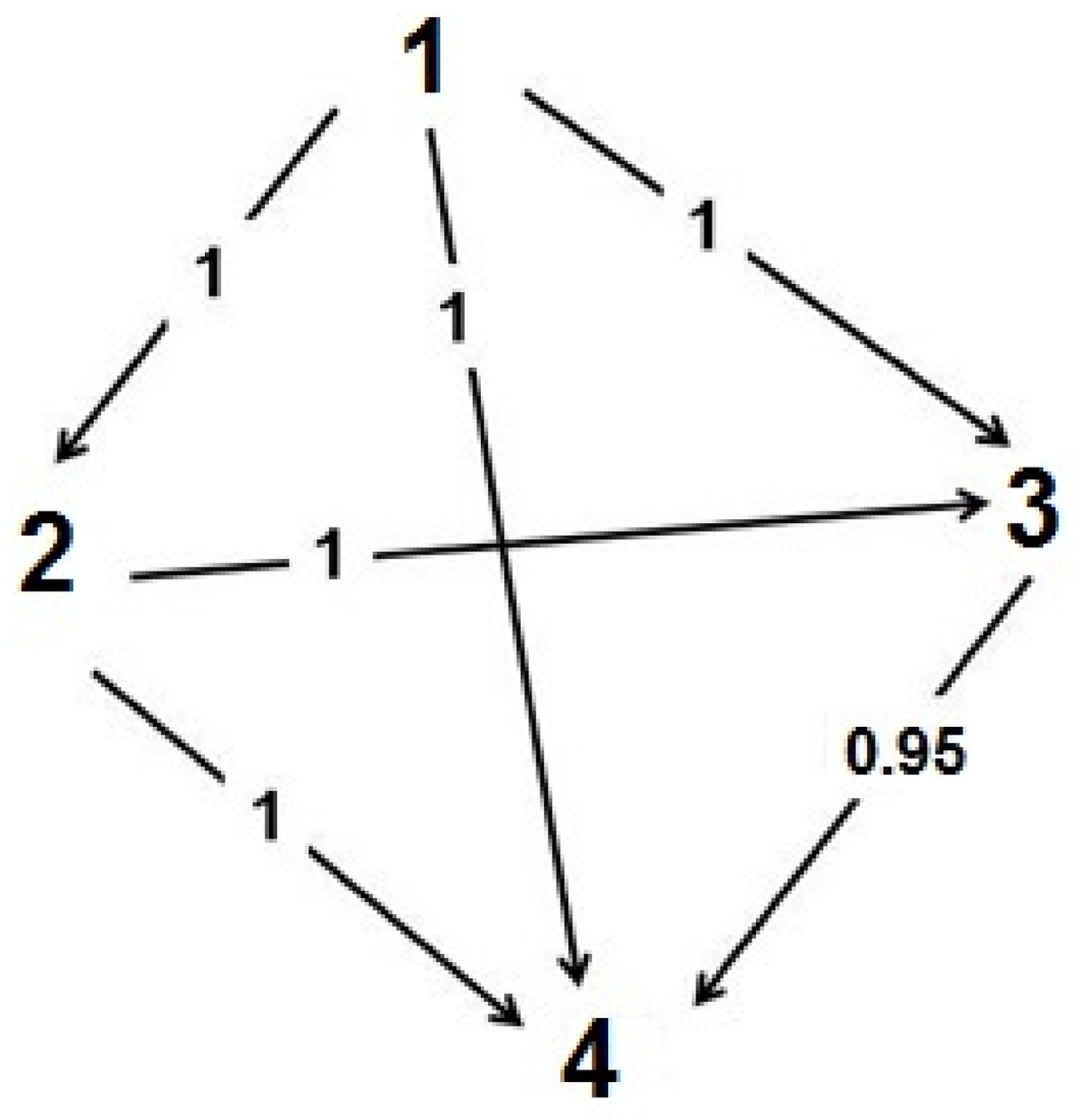
Dominance hierarchies in Andean condor (*Vultur gryphus*) feeding groups, according to pigmentation of individuals (N = 178 aggression interactions). Arrows indicate the direction of dominance from the dominant individual toward the subordinate one. Values on arrows indicate likelihood of success and between parenthesis the number of interactions between categories (N).

We were able to identify the pigmentation of individuals in 428 interactions. Pigmented individuals were observed in almost all age and sex classes, except for juvenile females, which did not show any degree of pigmentation. Adult males were the only individuals that reached the category of highest pigmentation (Table 2). We found that the hierarchy order in Andean condor feeding groups was associated with the degree of individual pigmentation (178 aggression interactions). In particular, the most pigmented individuals attacked all other categories and were constantly successful in all fights (High versus Medium, p<0.0001; High versus Low, p = 0.0005; High versus Unpigmented, p<0.0001). In turn, those individuals with medium level of pigmentation successfully confronted those with low pigmentation and those unpigmented (p = 0.0002 and p<0.0001, respectively). Finally, individuals with medium pigmentation always won fights against those which presented no pigmentation (p<0.0001) (Fig 5). In addition, taking into account the fights in which the opponents had the same attributes of sex and age, it was corroborated that the likelihood of success in fights effectively depends on the pigmentation of condors (n = 57, p = 0.0006).

**Table 2:** Percentage of pigmented Andean condor individuals for each pigmentation category by age and sex.

Age/sex category	Pigmentation level			
	High	Medium	Low	Unpigmented
Adult male	27.31	18.08	22.51	32.10
Sub-adult male	0.00	0.00	42.86	57.14
Juvenile male	0.00	4.55	18.18	77.27
Adult female	0.00	8.70	45.65	45.65
Sub-adult female	0.00	0.00	5.56	94.44
Juvenile female	0.00	0.00	0.00	100

## Discussion

As expected, we found that agonistic interactions between condors feeding at carcasses were related to the sex and the age categories, but interestingly also to their pigmentation. Adult males are major aggressors when feeding since they displace all individuals within the group, dominating all other categories. Possibly, this is due to the sex-specific differences in body size that drive dominance hierarchies in accessing resources [22], since the male condor can be up to 50% heavier than females [9], [18]. Sexual dimorphism in the Andean Condor is also responsible for partial temporal segregation between sexes to reduce agonistic encounters, which could lead to females programming routines that are not efficient in terms of energy, undertaking greater risks at feeding sites [9], [22]. The pattern of hierarchies dominated by size has been well observed in other social foraging species with marked sexual dimorphism (e.g. [36], [37]). In turn, the dominance relationship is clearer in female than in male Andean condors, and is determined by age. In this sense, juvenile females are completely subordinated to the other female categories (and also by males in general), and are expected to have a lower food intake according to the phenotypic limitation model [5].

Dominance relationships in the middle levels seem less clear compared to the extremes within a dominance hierarchy. These inconsistencies relative to the positions of dominance occupied by individuals [38] would lead to an incomplete linearity within the hierarchy. Attributes such as sex, age and pigmentation would be influencing foraging hierarchies at carcasses, creating different social dynamics at certain hierarchical levels, such as middle levels. Alternatively, coexistence of different personalities between individuals [39] may play a role in shaping dominance hierarchies. However, to fully understand how these foraging patterns are linked to population social structure, monitoring of a banded population would be required.

This study supports the idea that Andean condor pigmentation would also be a sign of social status *per se* since it reflects competitive superiority, regardless of the sex and age of individuals. This is possibly because the level of carotenoids in plasma of the Andean condor not only depends on age and sex but also on other factors related to the particular life-history attributes of this species [20] and, probably, also relates to the quality of each individual [10]. In addition, it is likely that the Andean condor will experience mechanisms associated with the variation in facial flushing between individuals of the same age; similarly as in the dominant individuals of the Lappet-faced vulture species *Aegypius tracheliotos* which are able to show this signal [13], [17].

Regardless of the physiological mechanism involved in the expression of pigmentation, the advantage of having this attribute is clear and corresponds to a greater likelihood of success in accessing food. Possibly, the difference in coloration allows a quicker resolution of conflicts, preventing major physical injuries in aggressors who fight for the dominant position in accessing food. This possible function of pigmentation has also been suggested for other social groups of scavengers and raptors [14], [40]. Likewise, it has been suggested that dominance is not always related to access to food, taking into account another type of benefit such as obtaining a partner by the brightest individuals who win an agonistic interaction [17].

Andean Condor juvenile females did not exhibit carotenoid-dependent pigmentation and, simultaneously, were completely subordinated by the other categories. This could be happening because producing carotenoid-dependent signals is more expensive mainly when individuals are under stress and / or with high activity of their immune and / or reproductive systems [10], [20], [40]. In a similar way, sustaining the facial flushing signal also represents a process that should be energetically expensive and potentially harmful to the body [13]. Consequently, under this idea, pigmentation would be manifesting itself as an "honest signal" [41]. Complementary studies will improve the understanding of whether investment in pigmentation can be inhibited by physiological and/or social mechanisms.

Reintroduction or reinforcement of wild Andean condor populations is a common strategy within conservation programs, which would be more effective if the sex ratio of released individuals and the recipient population were taken into account [42]. The results obtained in this study suggest that it is also necessary to consider the pigmentation levels of captive and rehabilitated individuals, due to their role in health and in the hierarchical rank they will occupy in the case of being released. For this, it is important to carry out further studies on the consistency of hierarchies based on carotenoid-dependent pigmentation and highlighted by facial flushing, which would imply the maintenance of pigmentation patterns by the same individual (even after being released) under different environmental circumstances and internal constraints.

In sum, this study corroborates a strong despotism in the Andean condor, with clear differences at opposite ends of the hierarchies, depending on sex and age. However, it demonstrates for the first time that pigmentation is also an attribute that corresponds with the social status of the Andean condor. Likewise, this evidence would show a possible adaptive value of pigmentation and a greater complexity of the hierarchy systems in this markedly sexually dimorphic and highly despotic species.

## Supporting information

S1 Table Interactions registered in the food groups of Andean condor feeding groups according to sex, age and pigmentation of individuals. (XLSX)
